# Angiotensin II accelerates mammary gland development independently of high blood pressure in pregnancy-associated hypertensive mice

**DOI:** 10.14814/phy2.12542

**Published:** 2015-09-04

**Authors:** Kazuya Murata, Altansarnai Baasanjav, Chulwon Kwon, Misuzu Hashimoto, Junji Ishida, Akiyoshi Fukamizu

**Affiliations:** 1 Life Science Center, Tsukuba Advanced Research Alliance, University of TsukubaTsukuba, Ibaraki, Japan; 2 Graduate School of Life and Environmental Sciences, University of TsukubaTsukuba, Ibaraki, Japan; 3 Ph.D. Program in Human Biology, School of Integrative Global Majors (SIGMA), University of TsukubaTsukuba, Ibaraki, Japan

**Keywords:** Angiotensin II, hypertension, mammary gland development, pregnancy

## Abstract

Angiotensin II (AngII) is a vasopressor hormone that has critical roles in maintenance of normal blood pressure and pathogenesis of cardiovascular diseases. We previously generated pregnancy-associated hypertensive (PAH) mice by mating female human angiotensinogen transgenic mice with male human renin transgenic mice. PAH mice exhibit hypertension in late pregnancy by overproducing AngII. A recent study demonstrated that angiotensin II type I (AT1) receptor is expressed in mammary epithelial cells and its signaling is critical for mammary gland involution after weaning. However, the role of AngII-AT1 receptor signaling in the development of mammary gland during pregnancy remains unclear. In this study, to investigate the role of AngII-AT1 receptor signaling in mammary gland development during pregnancy, we analyzed the mammary gland of PAH mice. Histological and gene expression analyses revealed that lobuloalveolar development was accelerated with increased milk protein production and lipid accumulation in the mammary gland of PAH mice. Furthermore, AT1 receptor blocker treatment suppressed acceleration of mammary gland development in PAH mice, while the treatment of hydralazine, another antihypertensive drug, did not. These data suggest that AngII-AT1 receptor-induced signaling accelerates mammary gland development during pregnancy through hypertension-independent mechanism.

## Introduction

The renin–angiotensin system (RAS) is an important endocrine pathway and plays an integral role in regulating blood pressure (Zaman et al. 2002). Angiotensin II (AngII), a major bioactive product of RAS, is produced through two-step cleavage of angiotensinogen by renin and angiotensin converting enzyme (ACE). AngII peptide induces vasoconstriction via activation of angiotensin II type 1 (AT1) receptor in vasculature (Zhou et al. 2003). Since deletion of RAS component gene, such as angiotensinogen, ACE and AT1a, results in severe hypotension in mice, it is widely accepted that the AngII-AT1 receptor signaling is necessary to maintain normal blood pressure (Tanimoto et al. 1994; Krege et al. 1995; Sugaya et al. 1995). In addition, RAS plays critical roles in a wide variety of biological processes including the development of cardiovascular diseases, drinking behavior, energy metabolism, and muscle regeneration (Weisinger et al. 1999; Kouyama et al. 2005; Ferrario 2006; Yoshida et al. 2013).

Mammary gland is the essential organ to produce milk and breastfeed pups in mammals. Mammary epithelial cells (MECs) form lobuloalveolar structure and begin to synthesize milk in pregnancy, and milk is continuously produced during lactation (Watson et al. 2011). After weaning, the mammary gland of dams regresses and returns to a nonpregnant state, which is called mammary involution (Watson et al. 2011). It has been reported that AT1 receptor is expressed in mammary gland (Inwang et al. 1997; Tahmasebi et al. 2006). Furthermore, a recent study has demonstrated that deletion of AT1 receptor gene or pharmacological blockade of AT1 receptor reduces activity of matrix metalloproteases and apoptosis of MECs in the mammary gland of mice, and thus mammary involution was delayed (Nahmod et al. 2012). However, the function of RAS in mammary gland development during pregnancy remains unclear.

We previously generated pregnancy-associated hypertensive (PAH) mice, which is pregnant human angiotensinogen transgenic (hANG) mice mated with male human renin transgenic (hRN) mice (Takimoto et al. 1996). Although hANG and hRN mice show normal blood pressure because of species specificity in the reaction between angiotensinogen and renin, feto-placental human renin flows into maternal circulation and react with human angiotensinogen in PAH mice (Fukamizu et al. 1993; Takimoto et al. 1996). Consequently, a large amount of AngII is produced and PAH mice exhibit maternal hypertension, cardiac hypertrophy, proteinuria, and intrauterine growth retardation of embryos during late pregnancy (Takimoto et al. 1996; Saito et al. 2004).

In this report, we investigate the role of AngII in mammary gland development during late pregnancy by analyzing PAH mice. We found that PAH mice displayed precocious lobuloalveolar structure and increased milk production. Moreover, AT1 receptor blocker treatment reversed these phenotypes, but another antihypertensive drug did not. These results suggest that AngII-AT1 receptor signaling has a promoting effect on mammary gland development during late pregnancy.

## Materials and Methods

### Animals

hANG and hRN mice were previously generated in our laboratory (Fukamizu et al. 1989; Takahashi et al. 1991). hANG females were mated with hRN males to create PAH mice. The day of mating was defined as day 0 of pregnancy (P0). PAH mice were treated with olmesartan or hydralazine from afternoon of P13 to P19. Olmesartan (15 mg/L, kindly gifted from Daiichi Sankyo, Tokyo, Japan) was administrated as described previously (Sakairi et al. 2008). Hydralazine hydrochloride (151278, MP Biomedicals, Santa Ana, CA) was administrated in drinking water. The dose of hydralazine was gradually increased to sufficiently suppress hypertension (62.5, 250, 375 mg/L). Hydralazine solution was changed at P15 and P17, respectively. C57BL/6J mice were used as control (wild-type [WT] mice, purchased from CLEA Japan, Tokyo, Japan). All animal experiments were carried out in a humane manner and approved by the Institutional Animal Experiment Committee of the University of Tsukuba. Experiments were conducted in accordance with the Regulation of Animal Experiments of the University of Tsukuba and the Fundamental Guidelines for Proper Conduct of Animal Experiments and Related Activities in Academic Research Institutions under the jurisdiction of the Ministry of Education, Culture, Sports, Science and Technology of Japan.

### Blood pressure measurement

Systolic blood pressure (SBP) of conscious mice was measured by tail-cuff method (BP98a, Softron, Tokyo, Japan) as described previously (Fukamizu et al. 1993; Takimoto et al. 1996; Saito et al. 2004; Sakairi et al. 2008; Ishimaru et al. 2012). SBP measurement was performed in the morning of P1, P3, P6, P9, and from P12 to P19.

### Whole mount carmine alum staining

Mammary glands were excised, spread on glass slides, and fixed in Carnoy’s fixative (60% ethanol, 30% chloroform, and 10% acetic acid) at room temperature for 6 h. After washing in 70% ethanol and distilled water, mammary glands were stained overnight at room temperature in carmine alum solution (0.2% carmine and 0.5% aluminum potassium sulfate). Glands were dehydrated in 70%, 95%, and 100% ethanol series, cleared in xylene and mounted with Entellan new (EMD Millipore, Billerica, MA). Images were acquired using SZ61 microscope (low magnification), BX53 microscope (high magnification), and DP21 digital camera (Olympus, Tokyo, Japan).

### Hematoxylin–eosin staining and immunohistochemistry

Mammary tissues were fixed for 24 h at room temperature in 10% formalin neutral buffer solution (062-01661, Wako, Osaka, Japan), embedded in paraffin and cut into 5-μm sections using a rotary microtome (HM340E; Microm International GmBH, Walldorf, Germany). Sections were deparaffinized, rehydrated, and stained with hematoxylin–eosin using standard techniques. For the detection of milk proteins, deparaffinized and rehydrated sections were treated with phosphate-buffered saline (PBS) containing 3% hydrogen peroxide to inactivate an endogenous peroxidase. Sections were blocked with G-block solution (GB-01, GenoStaff, Tokyo, Japan) and incubated with rabbit anti-mouse milk specific proteins antiserum (at 1:10,000 dilution, RAM/MSP, Nordic-MUbio, BC Susteren, the Netherlands) for 60 min. Sections were then treated with biotinylated anti-rabbit IgG antibody (at 1:200 dilution, BA-1000, Vector Laboratories, Burlingame, CA) for 30 min, washed in PBS, and incubated with horseradish peroxidase (HRP)-conjugated streptavidin (NEL750, PerkinElmer, Waltham, CA) for 30 min. To detect HRP activity, diaminobenzidine (D5905, Sigma, St. Louis, MO) was used as chromogenic substrate. Nuclei were counterstained with methyl green (138-12701, Wako). Bright field images were acquired using BX53 microscope and DP21 digital camera (Olympus). For adipophilin (ADFP) immunostaining, sections were blocked with tyramide signal amplification (TSA) blocking reagent (FP1020, PerkinElmer) and incubated with rabbit anti-ADFP antibody (at 1:100 dilution, ab108323, abcam, Cambridge, UK) for 60 min. After the incubation of biotinylated anti-rabbit IgG (426011, Nichirei Biosciences, Tokyo, Japan) for 30 min, antibodies were detected by TSA plus fluorescein system (NEL756001KT, PerkinElmer) according to the manufacturer’s protocol. Nuclei were counterstained with Hoechst 33258 (020-07844, Wako). Fluorescence images were obtained using confocal laser scanning microscope (FLUOVIEW FV10i, Olympus). Sizes of cytoplasmic lipid droplets (CLDs) were quantified using ImageJ.

### Quantitative real-time PCR

Mammary glands were harvested, flash-frozen in liquid nitrogen, and stored at –80°C until use. After the grinding of frozen mammary glands by Multi-beads shocker (Yasui Kikai, Osaka, Japan), total RNA was extracted using ISOGEN II (311-07361, NIPPON GENE, Tokyo, Japan) according to the manufacturer’s instruction. Total RNA was treated with DNase (M6101, Promega, Madison, WI) and reverse-transcribed using ReverTra Ace (TRT-101, Toyobo, Osaka, Japan). Real-time PCR was carried out using Thermal Cycler Dice Real Time System (Takara Bio, Shiga, Japan) with SYBR Green PCR master mix (Takara). Relative gene expression levels were evaluated using ΔΔCt method. Glyceraldehyde-3-phosphate dehydrogenase (GAPDH) gene was used as normalizer. Primer sequences are listed below.

*Csn2*: forward 5′-CTGCCACTCCACAACATTCC-3′,

*Csn2*: reverse 5′-GCATGATCCAAAGGTGAAAAGA-3′.

*Wap*: forward 5′-AGCCTTGTTCTTGGCCTGCT-3′,

*Wap*: reverse 5′-GGCACACTCCTCGTTGGTTT-3′.

*Xdh*: forward 5′-GTGGCAGACATCCCTTCCTG-3′,

*Xdh*: reverse 5′-CCTCACTGTTCCCGCCATT-3′.

*Btn1a1*: forward 5′-GCCAGGGGAAGGAAGTAGAG-3′,

*Btn1a1*: reverse 5′-ATGGACCCAATGGTGAGAAA-3′.

*Gapdh*: forward 5′-CTTTGGCATTGTGGAAGGGC-3′,

*Gapdh*: reverse 5′-CAGGGATGATGTTCTGGGCA-3′.

### Statistical analysis

Statistical analysis was performed using GraphPad Prism 5 (GraphPad Prism Software, La Jolla, CA). The data were analyzed with two-way analysis of variance (ANOVA) followed by Bonferroni multiple comparison test, Student’s *t*-test, and one-way ANOVA with Bonferroni posttest. Significant differences were defined as *P *<* *0.05.

## Results

### Changes in lobuloalveolar structure in PAH mice

**Figure 1:**
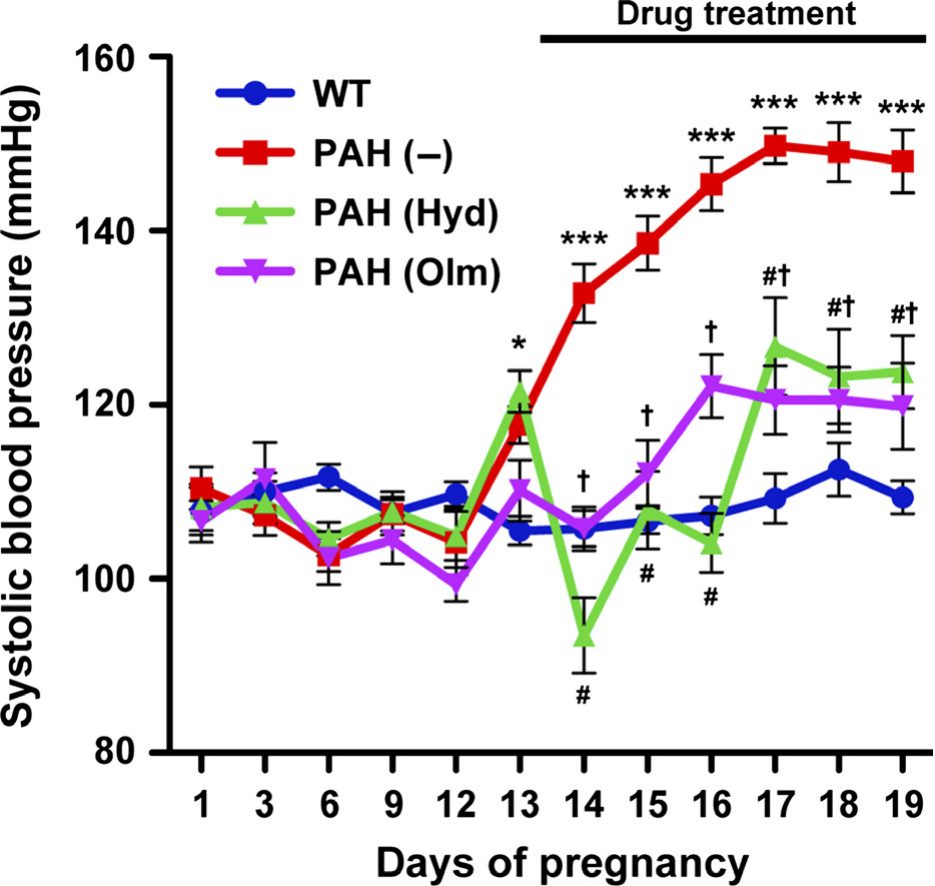
Alterations in maternal systolic blood pressure (SBP) during pregnancy. SBP was measured in wild-type (WT) (blue line), nontreated pregnancy-associated hypertensive (PAH) (red line), hydralazine (Hyd)-treated PAH (green line), and olmesartan (Olm)-treated PAH (purple line) mice. The data are presented as mean ± SEM, *n* = 4–13 mice per group. Data were analyzed with two-way analysis of variance (ANOVA) followed by Bonferroni multiple comparison test. *P <* *0.05, ***P <* *0.001 PAH (–) versus WT mice; ^#^*P *<* *0.001 PAH (Hyd) versus PAH (–) mice; ^†^*P *<* *0.001 PAH (Olm) versus PAH (–) mice.

To generate PAH mice, we crossed female hANG mice with male hRN mice. Consistent with previous studies (Takimoto et al. 1996; Saito et al. 2004; Sakairi et al. 2008; Ishimaru et al. 2012), SBP of PAH mice started to increase at day 13 of pregnancy (P13) and was significantly higher than that of WT mice during late pregnancy (Fig. 1).

**Figure 2:**
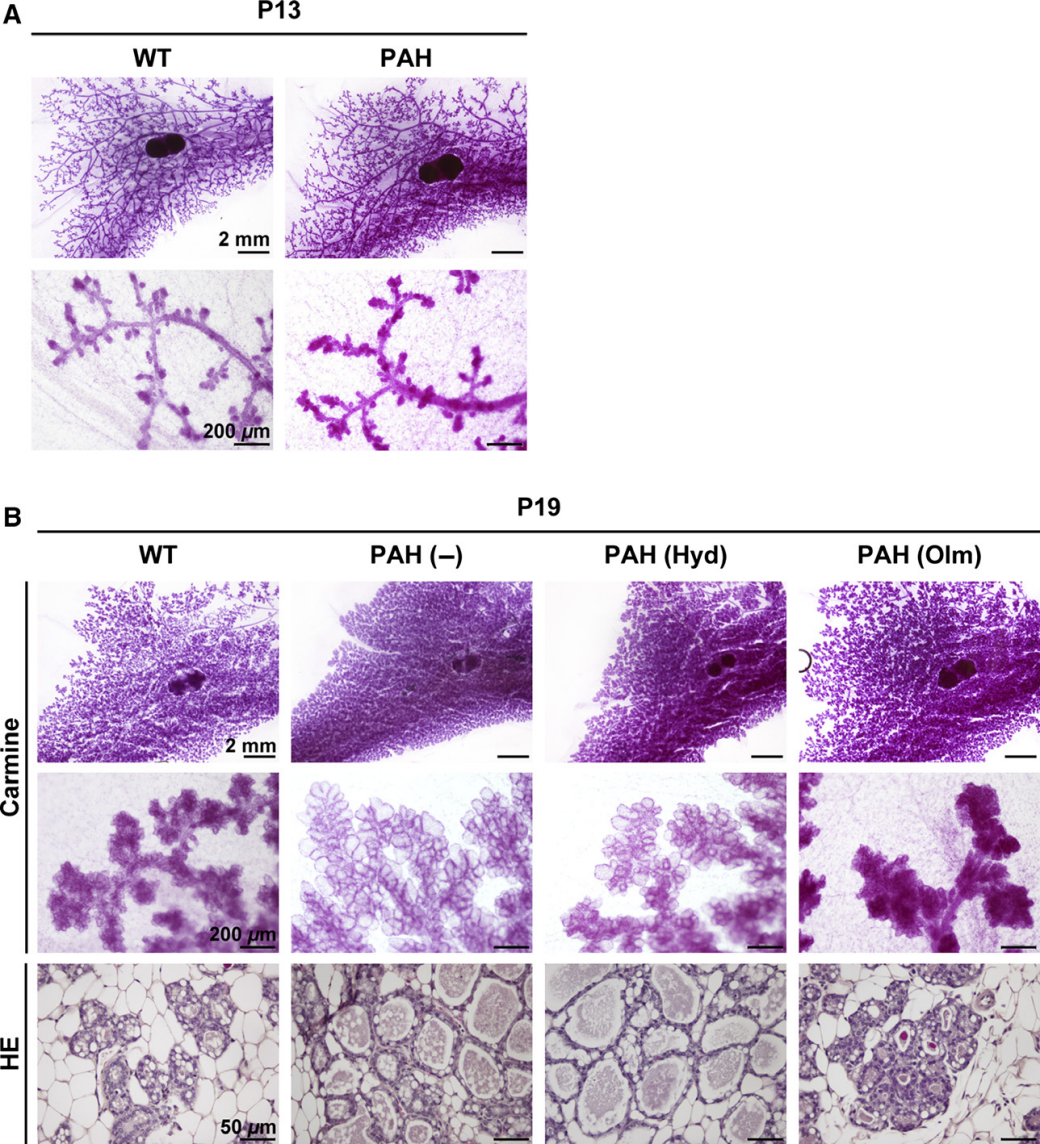
Lobuloalveolar structure of mammary gland in pregnancy-associated hypertensive (PAH) mice. (A) Representative images of mammary gland stained with carmine at P13 (*n* = 4 mice per group). Upper panels are low magnification images and lower panels are high magnification images. (B) Representative images of mammary gland stained with carmine and hematoxylin–eosin (HE) at P19 (*n* = 5 mice per group). Upper panels are low magnification images of whole mount mammary gland stained with carmine alum and middle panels are high magnification images. Bottom panels show mammary gland section stained with HE.

To evaluate the development of mammary gland in PAH mice during pregnancy, we first investigated lobuloalveolar structure of mammary gland by whole mount carmine staining and hematoxylin–eosin staining. No obvious differences were observed in lobuloalveolar structure between WT and PAH mice at P13 (Fig. 2A). In contrast, at P19, the lumina of lobuloalveoli were expanded in PAH mice compared with WT mice (Fig. 2B). These data suggest that PAH mice show precocious lobuloalveolar development during late pregnancy, because it is known that the dilation of lobuloalveolar lumen is a feature of lactating mammary gland.

### Increased milk protein and milk lipid accumulation in PAH mice during late pregnancy

**Figure 3:**
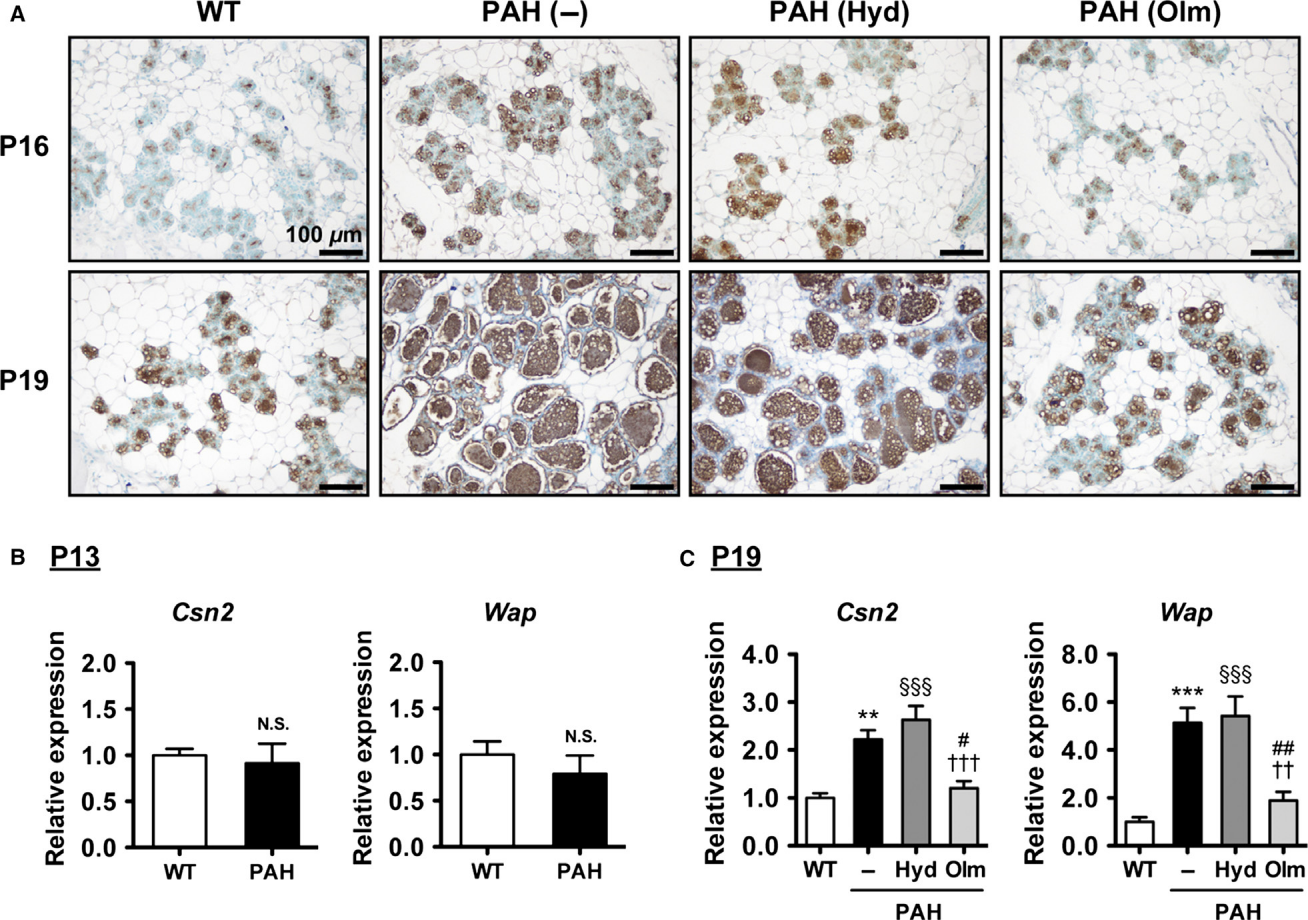
Milk protein production in the mammary gland of pregnancy-associated hypertensive (PAH) mice. (A) Immunostaining for milk proteins (brown) in the mammary gland at P16 and P19. Nuclei were counterstained with methyl green. (B, C) Gene expression levels of milk proteins (*Csn2* and *Wap*) determined by quantitative real-time PCR in the mammary gland at P13 (*n* = 4 mice per group) and P19 (*n* = 5 mice per group). The data are presented as mean ± SEM. Data were analyzed by Student’s *t*-test (B) and one-way analysis of variance (ANOVA) with Bonferroni posttest (C). **P <* *0.01, ***P <* *0.001 PAH (–) versus WT mice; ^§§§^*P *<* *0.001 PAH (Hyd) versus WT mice; ^#^*P *<* *0.05, ^##^*P *<* *0.01 PAH (Olm) versus PAH (–); ^††^*P *<* *0.01, ^†††^*P *<* *0.001 PAH (Olm) versus PAH (Hyd) mice. N.S. indicates not significant. WT, wild-type; Hyd, hydralazine treated; Olm, olmesartan treated.

During pregnancy, mammary gland begins to express milk protein and accumulate milk lipid to produce sufficient amount of milk for neonates. We investigated milk protein expression in the mammary gland of PAH mice by immunohistochemistry and gene expression analysis. Immunostaining for milk proteins showed that milk protein-positive areas were increased in the mammary gland of PAH mice compared with WT mice at P16 and P19 (Fig. 3A). Furthermore, gene expression levels of β-casein and whey acidic protein (*Csn2* and *Wap*), which are the major milk proteins, were significantly elevated in the mammary gland of PAH mice compared with those of WT mice at P19 (Fig. 3C), whereas there are no differences in expression levels between WT and PAH mice at P13 (Fig. 3B). These results demonstrate that milk protein synthesis is increased in the mammary gland of PAH mice.

**Figure 4:**
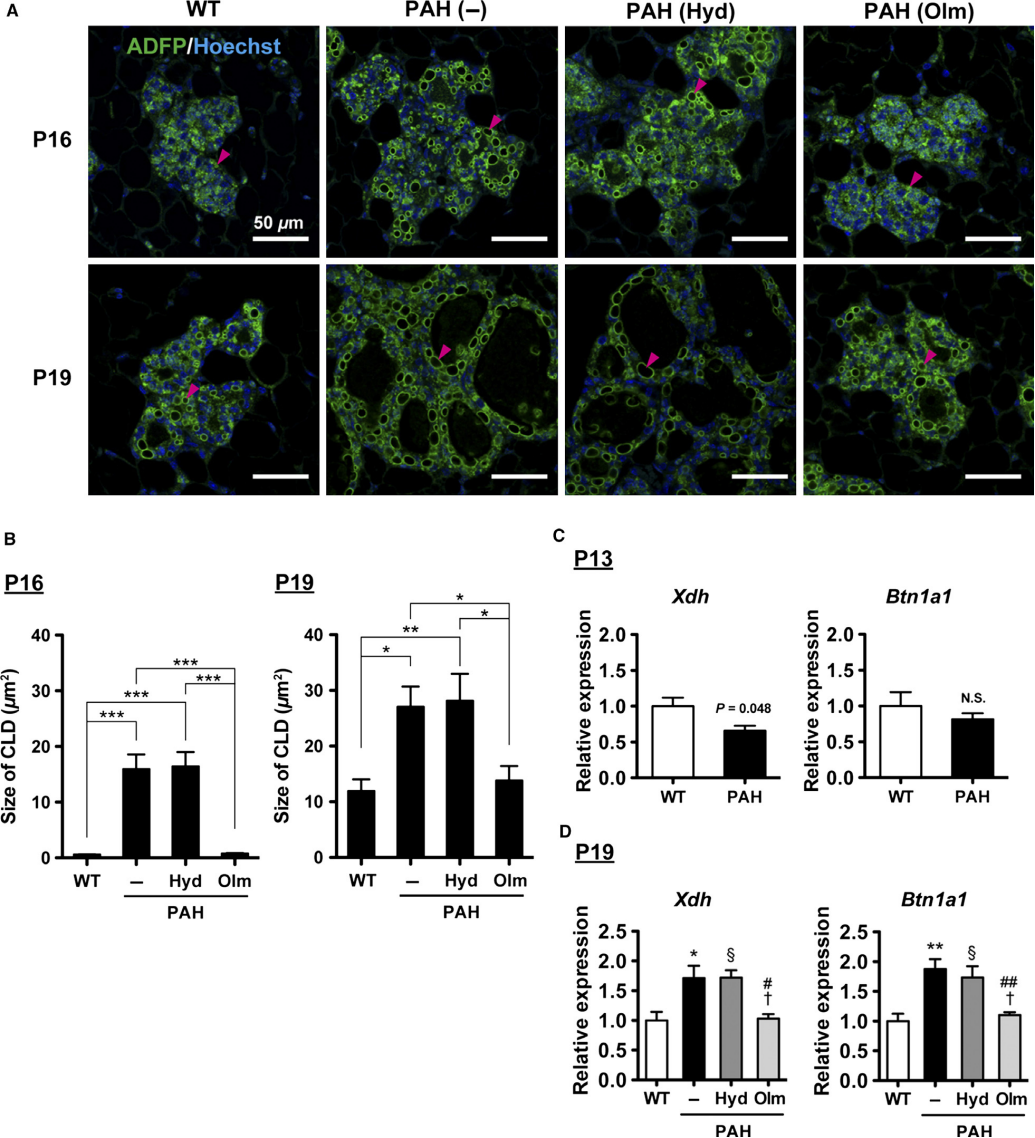
Milk lipid accumulation in the mammary gland of pregnancy-associated hypertensive (PAH) mice. (A) Representative images of immunostaining for adipophilin/perilipin 2 (ADFP) protein (green) at P16 and P19. Arrowheads indicate cytoplasmic lipid droplets (CLDs). (B) Quantification of CLD size. The data are presented as mean ± SEM, *n* = 50 CLDs per group. Data were analyzed by one-way analysis of variance (ANOVA) with Bonferroni posttest. *P < 0.05, **P <* *0.01, ***P < 0.001. (C, D) mRNA levels of *Xdh* and *Btn1a1* determined by quantitative real-time PCR in the mammary gland at P13 (*n* = 4 mice per group) and P19 (*n* = 5 mice per group). The data are presented as mean ± SEM. Data were analyzed by Student’s *t*-test (C) and one-way ANOVA with Bonferroni posttest (D). *P <* *0.05, **P <* *0.01 PAH (–) versus WT mice; ^§^*P *<* *0.05 PAH (Hyd) versus WT mice; ^#^*P *<* *0.05, ^##^*P *<* *0.01 PAH (Olm) versus PAH (–); ^†^*P *<* *0.05 PAH (Olm) versus PAH (Hyd) mice. N.S. indicates not significant. WT, wild-type; Hyd, hydralazine treated; Olm, olmesartan treated.

Milk lipids are supplied by secretion of CLD from luminal MECs (Chong et al. 2011). In late pregnancy, CLDs accumulate and enlarge in luminal MECs. It has been reported that adipophilin/perilipin 2 (ADFP) is a CLD binding protein and regulates maturation of CLD (Russell et al. 2011). To assess milk lipid accumulation, we visualized CLDs by immunostaining for ADFP protein and compared the sizes of CLDs. At P16, large CLDs were observed in the MECs of PAH mice, while only dot-like small CLDs existed in WT mice (Fig. 4A and B). Although large CLDs appeared in MECs of WT mice at P19, CLDs in MECs of PAH mice were significantly larger than that of WT mice (Fig. 4A and B). These data show that milk lipid accumulation is elevated in the mammary gland of PAH mice. We further investigated the expression levels of genes, which are required for CLDs secretion from MECs to lumen. It has been demonstrated that *Xdh* and *Btn1a1* are essential genes for CLD secretion, because deletion of these genes causes defect of CLD secretion in the murine mammary gland (Vorbach et al. 2002; Ogg et al. 2004). At P13, there was no significant increase in *Xdh* and *Btn1a1* mRNA levels in PAH mice compared with WT mice (Fig. 4C). In contrast, the expression levels of *Xdh* and *Btn1a1* were significantly increased in PAH mice compared to WT mice at P19 (Fig. 4D). Taken together, the above data strongly suggest that PAH mice exhibit accelerated mammary gland development and excessive AngII promotes mammary gland development during late pregnancy.

### High blood pressure is not required for accelerated mammary gland development in PAH mice

It has been reported that hypertension induces cellular responses in several organs, such as cardiovascular tissues, cerebral tissue, and retina, in a RAS-independent manner (Silva et al. 2007; Hermes et al. 2008; Westhoff et al. 2008). However, the relationship between hypertension and mammary gland development during pregnancy is unknown. Therefore, we next investigated the effect of antihypertensive drugs on mammary gland development in PAH mice.

At first, we treated PAH mice with hydralazine, which is thought to be a RAS-independent vasodilator. SBP was significantly reduced by hydralazine treatment in PAH mice (Fig. 1), in which lobuloalveolar structure was comparable to that of nontreated PAH mice at P19 (Fig. 2B). Furthermore, hydralazine treatment did not affect milk protein expression levels in PAH mice (Fig. 3A and C), and the sizes of CLDs in hydralazine-treated PAH mice were similar to nontreated PAH mice at P16 and P19 (Fig. 4A and B). There were no significant differences in *Xdh* and *Btn1a1* gene expression levels between nontreated and hydralazine-treated PAH mice (Fig. 4D). These data suggest that hypertension does not affect mammary gland development in PAH mice.

### AngII-AT1 signaling accelerates mammary gland development in PAH mice

Next, we administered another antihypertensive drug, olmesartan, a selective antagonist of AT1 receptor, to PAH mice. Olmesartan treatment significantly suppressed SBP of PAH mice as well as hydralazine treatment (Fig. 1). Interestingly, as shown in Figure 2B, lobuloalveoli of olmesartan-treated PAH mice were smaller than those of non- and hydralazine-treated PAH mice and were similar to those of WT mice at P19. Immunohistochemical analysis revealed that olmesartan treatment resulted in decreased milk protein-positive areas in mammary gland of PAH mice at P16 and P19 (Fig. 3A). The mRNA levels of *Csn2* and *Wap* were significantly lower in olmesartan-treated PAH mice than those of nontreated PAH mice (Fig. 3C). The sizes of CLDs were significantly reduced in olmesartan-treated PAH mice compared with nontreated PAH mice at P16 and P19 (Fig. 4A and B). Olmesartan treatment significantly decreased *Xdh* and *Btn1a1* gene expression levels in PAH mice at P19 (Fig. 4D). These results indicate that activation of AT1 receptor has critical roles in accelerating mammary gland development in PAH mice.

## Discussion

Mammary gland development during pregnancy is a highly complex process regulated by multiple hormone–receptor signalings. In the present study, we have demonstrated that PAH mice exhibit accelerated mammary gland development in late pregnancy, and hypertension is not required for acceleration of mammary gland development. Importantly, AT1 receptor blocker treatment prevented precocious mammary gland development in PAH mice, indicating that AngII promotes mammary gland development during late pregnancy through activation of AT1 receptor.

We have previously reported that the body weight gain of foster pups nursed by PAH mice is reduced compared with foster pups nursed by WT mice (Murata et al. 2013). It has been shown that the diminished mammary gland development during pregnancy in mothers contributes to failure of lactation after parturition, and impairs growth of their offspring (Mulac-Jericevic et al. 2003). Therefore, we initially predicted that the development of mammary gland was impaired in PAH dams. However, unexpectedly, we found here that mammary gland development during pregnancy is accelerated in PAH mice. This discrepancy might be explained by following reasons. Although maternal hypertension in PAH mice is improved in postpartum period by reduction of AngII levels, high blood pressure is observed within 2 days after parturition (Takimoto et al. 1996), indicating that AngII remains in maternal circulation during early postpartum period. Therefore, this raises the possibility that AngII has potentials for suppression of milk ejection or frequency of breast feeding in PAH mice, resulting in growth retardation of their foster pups.

In this work, we did not address how AngII-AT1 signaling regulated mammary gland development in PAH mice. Lobuloalveolar development and milk production is stimulated by a wide variety of endocrine hormones, including progesterone and prolactin (Ormandy et al. 1997; Lindeman et al. 2001; Mulac-Jericevic et al. 2003; Brisken and O’Malley 2010). Therefore, one possibility is that AngII activates AT1 receptor in endocrine tissues, such as ovary and pituitary gland, to modulate synthesis and/or secretion of these hormones. Consequently, hormones induced by AngII might accelerate mammary gland development. In addition, another is that AngII activates AT1 receptor in the mammary gland and directly promotes proliferation and functional differentiation of MECs, because it has been shown that AT1 receptor is expressed in human and mouse MECs (Inwang et al. 1997; Nahmod et al. 2012). Further investigation is needed to clarify the target tissue of AngII and molecular mechanisms.

Until now, few studies have investigated the relationship between RAS and mammary gland in vivo. Although a previous report has revealed that mice with deletion of both AT1a and AT1b genes, which are the subtype of AT1 receptor and encoded in other gene loci, can normally breastfeed their pups (Nahmod et al. 2012), we show stimulatory effect of AngII-AT1 signaling on mammary gland development during late pregnancy by analyzing our unique mouse model with pregnancy-associated hypertension. The present work uncovers the previously overlooked function of activated RAS in mammary gland development during pregnancy and provides helpful information to understand the role of RAS in the biology of mammary gland.
